# Explaining the experience of prenatal care and investigating the association between psychological factors with self-care in pregnant women during COVID-19 pandemic: a mixed method study protocol

**DOI:** 10.1186/s12978-020-00949-0

**Published:** 2020-06-18

**Authors:** Marzieh Masjoudi, Armin Aslani, Somayyeh Khazaeian, Azita Fathnezhad-Kazemi

**Affiliations:** 1 Department of Midwifery, Faculty of Nursing and Midwifery Islamic Azad University, Rasht branch, Rasht, Iran; 2 Student Research Committee, Islamic Azad University, Tabriz branch, Tabriz, Iran; 3 Pregnancy Health Center, Zahedan University of Medical Sciences, Zahedan, Iran; 4 Department of Midwifery, Faculty of Nursing and Midwifery Islamic Azad University, Tabriz branch, Tabriz, Iran

**Keywords:** Experiences, Prenatal care, COVID-19, Self-care, Fear, Anxiety, Perceived stress

## Abstract

**Background:**

Coronavirus disease 2019 (COVID-19) is a novel global public health emergency. Prenatal care (PNC) providing institutes should identify the needs and demands of pregnant women by optimizing the means of PNC services during the COVID-19 pandemic. The present study aims to: a) explain prenatal care experiences; b) assess the factors affecting self-care, and c) present a prenatal care guideline and Strategies to improve the PNC.

**Methods:**

This mixed-methods study with a sequential explanatory design consists of three phases. The first phase is a qualitative study exploring the prenatal care experiences among pregnant women. In this phase, the subjects will be selected through purposive sampling; moreover, in-depth individual interviewing will be used for data collection. Finally, the conventional content analysis approach will be employed for data analysis. The second phase is quantitative and will be used as a cross-sectional approach for assessing the association between psychological factors of self-care. In this regard, a multistage cluster sampling method will be used to select 215 subjects who will be visited in health care centers of Tabriz, Iran. The third phase will be focusing on developing a prenatal care guideline and Strategies, using the qualitative and quantitative results of the previous phases, a review of the related literature, and the nominal group technique will be performed among experts.

**Discussion:**

The present research is the first study to investigate the prenatal care experiences and factors influencing self-care among pregnant women during COVID-19 pandemic. For the purposes of the study, a mixed-methods approach will be used which aims to develop strategies for improving health care services. It is hoped that the strategy proposed in the current study could lead to improvements in this regard.

**Ethical code:**

IR.TBZMED.REC.1399.003.

## Plain English summary

Coronavirus disease 2019 (COVID-19) is a global public health emergency and a novel, highly contagious pneumonia caused by the severe acute respiratory syndrome coronavirus 2 (SARS-CoV-2). Pregnant women are more susceptible to viral infection due to pregnancy alterations. Social distancing program and fear of getting the sick lead to the reduction of prenatal care referrals, additionally pregnant women are also more willing to reduce the frequency of prenatal care. The lack of PNC during pregnancy might be drastically harmful. Prenatal care services institutes should identify the fears and demands of pregnant women during the COVID-19 pandemic by optimizing the means of PNC services and providing a service for the safety and well-being of pregnant women. The current study provides precise information about the prenatal care experiences of pregnant women, and the factors influencing it. This study will be used in the mixed methods approach with the sequential explanatory design and is comprised of three phases. The first phase is qualitative and will be exploring the experiences of pregnant women. The second phase is quantitative and will be used as a cross-sectional approach to assess the factors affecting self-care in pregnant women in Tabriz. The findings of the qualitative and quantitative phases, the literature review, and the nominal group technique will be used to establish some strategies to improve and may promote prenatal care.

## Introduction

The COVID- 19 disease is an emerging infection and also a re-emerging viral respiratory disease that has affected most countries in the world and its prevalence is increasing [1–3]. The virus is rapidly spreading around the world and due to the rapid spread of the virus, the World Health Organization (WHO) has identified it as a contagious pandemic disease [4]. Despite the strong efforts taken to control the epidemic, Nearly 5 million people worldwide are reported to have contracted the infection [5, 6]. The current state of the COVID-19 outbreak in all over the world is concerning [7, 8]. People over the age of 60 and those with weakened immune systems are at higher risk for the disease [9], and because the immune system is partially suppressed during pregnancy; pregnant women are more vulnerable to viral infections and their complications; thus COVID-19 pandemic might have serious consequences for pregnant women [1, 10]. The available data about effects of COVID- 19 on pregnancy and its probable complications seems to be insufficient [9, 11]. However, due to the physiological changes in maternal immune and cardiopulmonary systems, pregnant women are more likely to develop severe illness after infection with respiratory viruses [2, 10]. In 2009, pregnant women accounted for 1% of patients infected with influenza A subtype H1N1 virus, but they accounted for 5% of all H1N1-related deaths [12]. Severe complications have also been reported in SARS and MERS viruses epidemic, including tracheal intubation, hospitalization in the intensive care unit, renal failure, and death during pregnancy [13]. Reviews of studies have shown that clinical signs, laboratory results, and radiographic criteria in pregnant women with COVID-19 are similar to other affected adults, but the risk of developing COVID-19 disease in pregnant women is high for the reasons stated above and there is the possibility of complications in pregnancy and newborn infection [14]. These factors have led to anxiety and stress in pregnant women and their families [1, 15]. On the other hand, with the rapid spread of the disease in different countries, including Iran, taking special precautions like physical distancing and quarantining are recommended to protect and prevent the disease [16]. However, social isolation and disconnection can cause sadness, concerns, fear, anger, irritation and frustration [17]. All of factors that mentioned above are effective in self-care and health-care behaviors [1]. Such behaviors are performed by individuals to maintain and promote health level and disease prevention [18]. However, pregnant women have concerns about how to take care of their pregnancies and how to carry out their plans for childbirth during the outbreak of the novel corona virus [1]. Social distancing program and fear of getting the sick lead to the reduction of prenatal care referrals, additionally pregnant women are also more willing to reduce the frequency of prenatal care visits [8, 19]. The lack of PNC during pregnancy might be drastically harmful [8]. According to the reports; most of them are interested in getting online access to health information and services [8, 15]. Since qualitative studies can be helpful for a deep understanding of a particular phenomenon, and also, due to insufficient studies regarding prenatal care and its barriers or facilitators during COVID– 19 pandemics, present study was designed to explore factors affecting prenatal care. So appropriate interventions and guidelines can be developed in this regard.

### Objectives

The objectives of each phase are as follows:

### Objectives of the first phase: qualitative study

1. To explore prenatal care experiences among pregnant women during the COVID-19 pandemic.
2. To explore views about barriers and facilitators of prenatal care among pregnant women during the COVID-19 pandemic.

### Objectives of the second phase: quantitative study

1. To determine self-care behaviors among pregnant women during the COVID-19 pandemic.
2. To determine fear of COVID-19 among pregnant women during the COVID-19 pandemic.
3. To determine anxiety of COVID-19 among pregnant women during the COVID-19 pandemic.
4. To determine perceived stress among pregnant women during the COVID-19 pandemic.
5. To determine the association between fear of COVID-19 with self-care behaviors.
6. To determine the association between anxiety of COVID-19 with self-care behaviors.
7. To determine the association between perceived stress with self-care behaviors.

### Objectives of the third phase

1. To develop a prenatal care guideline and Strategies to improve the PNC and increase the self- care skills during the COVID-19 pandemic.

## Methods/design

### Study design

**Fig. 1:**
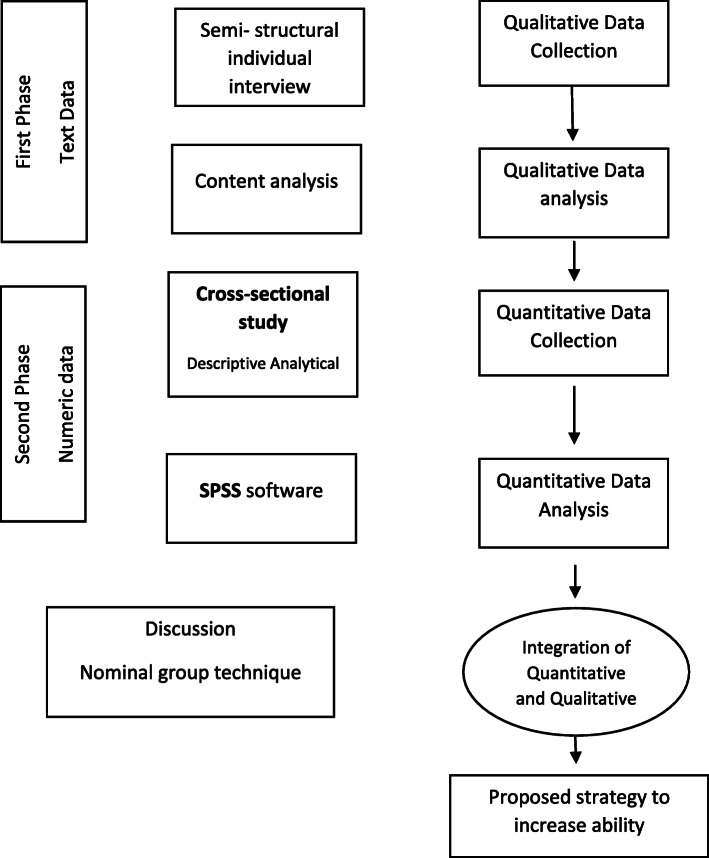
Study diagram

A mixed methods sequential explanatory design will be used to conduct this study by collecting, analyzing, and integrating the qualitative and quantitative data. The mixed-methods paradigm is based on the principles and logic of pragmatism. According to this paradigm, the mixed use of qualitative and quantitative approaches results in a better understanding of the problem [20]. This study will have three phases, and the qualitative and quantitative data will be collected in the first and second phases, respectively. Phase one is an exploratory qualitative study, which aims to explore prenatal care experiences among pregnant women during the COVID-19 pandemic. Phase two is a cross-sectional study, which aims to assess the factors affecting the self-care behaviors among pregnant women during the COVID-19 pandemic. Phase three is about developing an evidence based and a culturally sensitive guideline based on literature review, the results of phase one and two and experts’ opinion using the nominal group technique (Fig. 1).

### First phase: qualitative study

The first Phase is an exploratory qualitative study with a conventional content analysis approach to explore the prenatal care experiences and views about barriers and facilitators of prenatal care among pregnant women during the COVID-19 pandemic.

### Sampling method

The research participants will be selected through purposive sampling among pregnant women. Inclusion criteria included: having Iranian nationality and residency in Tabriz, ability to understand and to convey concepts to the researcher, single pregnancy with the age range of 18–40 years, pregnancy over 10 weeks, having the tendency and ability to express and transfer concepts, considering the maximum variability in terms of factors such as education, age, socioeconomic status, having any disease or medical, obstetric risk factor identified on the basis of the family’s health records or, as the pregnant woman claims, also requiring special care or relative or absolute rest for the mother, as well as the withdrawal and non-attendance of the pregnant women were considered as the exclusion criteria.

### Data collection

Qualitative data will be collected using in-depth and semi-structured interviews, containing open questions. Before conducting the interviews, the research team reviews the questions, and the ways to obtain valid data, then focuses on research questions. The interviewer will pilot the interview on with a subset of participants, and will use this information to further refine the guide with respect to culturally sensitive and appropriate questions. The interview will begin with a key question, “What were your experiences and feelings about prenatal care during the covid-19 pandemic?”, “Do you think the covid-19 pandemic has affected your prenatal care?”. Then, the interview will continue by presenting other questions, such as “How do you cope with the difficulties of prenatal care during the covid-19 pandemic?”, “What can you do to prevent the disease during pregnancy?”, “What do you think about prenatal care support?”, “Have you received support?”, “In your opinion What factors can be helpful in prenatal care?”, and “What factors do you think may hinder your prenatal care?”. The interview will continue with more in-depth items, such as “what do you mean? Why? Can you explain further? Can you give an example?” to explore the depth of their experience. During the interview, as far as possible, the Note field will be used and non-verbal data such as tone of voice and behaviors will be recorded, too. The sampling will continue until data are saturated. All interviews will be carried out in care centers in a quiet room and without someone other than the interviewees.

### Data analysis

Data analysis process will be conducted simultaneously with data collection using MAXQDA software version 10. Qualitative data analysis can be conducted through qualitative content analysis based on the Graneheim and Lundman method [20]. In this approach, the data will be analyzed through repeated Reading strategy to obtain a full understanding of it. Then, the texts will be read word by word to extract the codes. First, the objective words that contain the key concepts will be specified. The researcher continued digging the text by taking notes from the initial analysis until the major codes will be extracted. In this process, the code labels reflecting more than one key thought will be directly extracted and specified. Then, the codes will be categorized based on their difference and/or relationships. The codes will be categorized into themes and main categories. Subcategories will be extracted based on differences and similarities.

### Validation

To validate the results, at first efforts will be made to build a friendly relationship with the participants. In order to increase the accuracy of the data and to verify accuracy of the data, after the registration, the interviews will be given to the participants to review and confirm their stated content and, necessary points will be any other content, it will be added to the data. Interviews will frequently be read by the corresponding author of the paper; then, the text of the interviews with the extracted codes and categories will be shared with the colleagues and their comments will be used. External monitoring will be also used to increase the reliability. Through providing the initial code derived from the analysis and examples of the extraction, as external observers, the concepts will be given to other researchers; who are not related to the study in order to determine whether they also will have a similar perception of the data or not.

### Second phase: quantitative study

First, a cross-sectional descriptive analytical study will be conducted to evaluate association between psychological factors with self-care in pregnant women.

### Sample size and sampling method

Considering an acceptable error of 0.1 around the mean (m = 2.14), 95% confidence coefficient, 90% statistical power, and the standard deviation of 1.11 related to fear of COVID- 19 [21], the necessary sample size was determined to be 103 pregnant women, and regarding 95% confidence coefficient, 90% statistical power, an acceptable error of 0.05 around the mean (m = 71.9), and the standard deviation of 11.48 related to self-care, also an acceptable error of 0.05 around the mean (m = 60.27), and the standard deviation of 13.12 related perceived stress [18], the necessary sample size was determined to be 40 and 73 pregnant women, respectively. Also, Considering 57% anxiety with an acceptable error of 0.1 [22], the necessary sample size was determined to be 130. As the sample size based on anxiety was greater, this was the sample size sought. Regarding cluster sampling and design effect of 1.5, the final sample size was considered to be 215 pregnant women.

$$ n=\frac{{\left({Z}_{1-\frac{a}{2}}\right)}^2x\;p\left(1-p\right)}{d^2} $$

Sampling will be conducted in the healthcare centers in Tabriz. There are 82 public healthcare centers (*n* = 40) and posts (*n* = 42) in different regions of the city that provide primary care services, free of charge. Most of the pregnant women have health records in these healthcare centers and posts. A two-stage cluster sampling will be carried out so that 13 healthcare centers and 14 healthcare posts were selected using a randomizer software (www.random.org). A list of all pregnant women at each center will be extracted from the health records and the samples will be randomly selected from the ordered numbers. Afterward, using the phone number registered in each record, the researcher will call the potential participants and will invite them to participate in the study and will visit them in their homes if needed. Eligible participants will be provided with full explanations about the study objectives and procedures. Also, informed consent will be obtained before collecting information, and the importance of honest answers to the questionnaire will be emphasized, and they are asked to complete the anonymous questionnaires in a private room. A number of participants are likely to be illiterate, so the questionnaires will be completed by the researcher in order that the data collection method to be the same for all individuals.

### Inclusion criteria

The inclusion criteria consisted of 1) Iranian nationality, 2) residency in Tabriz, 3) ability to read and write, 4) intention to participate in the study, 5) single pregnant women with the age range of 18–40 years, 6) pregnancy over 10 weeks, 7) no history of stressors in the last 6 months (e.g., divorce, death of a family member, and diagnosis of a family member with an incurable or life-threatening disease), 8) Having any disease or medical, obstetric risk factor identified on the basis of the family’s health records or, as the pregnant woman claims, also requiring special care or relative or absolute rest for the mother.

### Exclusion criteria

The exclusion criteria were failure to complete the questionnaire completely and unwillingness to continue the study.

### Scales and data collection

Quantitative data will be collected using 5 questioners, including:

1. **Sociodemographic characteristics questionnaire:** consisted of questions about the women and their husband’s ages, women and their husband’s education, number of pregnancies, number of deliveries, gestational age, weigh, height, Number of prenatal care.

2- **Fear of COVID-19 Scale (FCV-19S):** This scale which is provided by Ahorsu and et al. [23], includes seven items. The participants indicate their level of agreement with the statements using a five-item Likert type scale. Answers included “strongly disagree,” “disagree,” “neither agree nor disagree,” “agree,” and “strongly agree”. The minimum score possible for each question is 1, and the maximum is 5. A total score is calculated by adding up each item score (ranging from 7 to 35). The higher the score, the greater the fear of coronavirus-19. More specifically, reliability values such as internal consistency (α = .82) and test–retest reliability (ICC = .72) were acceptable.
3- **Corona Disease Anxiety Scale (CDAS):** This questionnaire has been developed by Alipour et al. in Iran [24]. The final version of this scale has 18 items and 2 components. Items 2 to 8 measure psychological symptoms and items 9 to 18 measure physical symptoms. The instrument is rated on a 4-degree Likert scale (never = 0, sometimes = 1, most of the time = 2, and always = 3); Therefore, the highest and lowest scores obtained by the respondents in this questionnaire are between 0 and 54. Higher scores in this questionnaire indicate a higher levels of anxiety in individuals. The reliability of this tool was obtained using Cronbach’s alpha method for the cause of psychological symptoms (0.879), for physical symptoms (0.861) of the first questionnaire (0.919).
4- **Perceived Stress Scale (PSS):** which is provided by Cohen et al. [25] in 1983 with 3 versions of 4, 10, 14 that was applied for measuring perceived stress in past 1 month. We chose to use version 14 in this study. Each question has 5 options that half of them are direct (0, 1, 2, 3, 4), and the other half are reverse (4, 3, 2, 1, 0) scoring formats. All items are based on the Likert scale (0 = never, 1 = low, 2 = moderate, 3 = much, 4 = very much) scoring. Scores are ranged from 0 to 56 sets. It should be noted that 7 questions as positive concepts (4, 5, 6, 7, 9, 10, 13) are reverse (4 = never, 3 = little, 2 = moderate, much = 1, too much = 0). Reliability of PSS test was 0.85 and internal consistency of this test was calculated of 0.84 to 0.86 (43, 42). The homogeneity coefficients of this questionnaire in the Iranian population were also confirmed by Harris and Mousavi with the Cronbach’s alpha was 0.84 [26].
5- **Pregnancy Self-Care questionnaire:** It consists of 13 questions that are rated on a 4-degree Likert scale (never = 1, sometimes =2, most of the time = 3, and always = 4). The reliability of the questionnaire in the internal consistency method, Cronbach’s alpha is 0.85 [18].

### Data analysis

SPSS-22 software is used to analyze the quantitative data. Sociodemographic, FCV-19S, PSS, CDAS and scoring of Pregnancy Self-Care questionnaires score will be described by frequency (percent), as well as mean (standard deviation) if the data are normally distributed. The association between Sociodemographic, FCV-19S, PSS, CDAS with Pregnancy Self-Care will be determined using the independent test, ANOVA and Pearson correlation tests in the bivariate analysis, and logistic linear regression adjusting the confounding variables in the multivariate analysis.

### Third phase: integration of quantitative and qualitative data

To develop a prenatal care guideline and Strategies to improve the PNC and increase the self- care skills during the COVID-19 pandemic, a comprehensive literature review will be carried out with a supportive approach to improve such practices. Following this, the results from qualitative and quantitative studies will be delivered to 10–12 experts. Then, their feedback and comments will be taken into account, using the nominal group technique.

## Discussion

The current epidemic of COVID-19 is severe and worrying all over the world [27], and the number of pregnant women with the virus is on the rise because of pregnancy is a physiological state that predisposes women to viral infection [15, 27]. With the rise in global attention, much has been done to better understand and control COVID-19 from various perspectives [4]. Nonetheless, little has been mentioned regarding maternal health and prenatal care management [28]. Regarding the needs for health knowledge on COVID-19, personal protection against 2019 novel coronavirus (2019-nCoV) is the most concerned for pregnant women [13, 15]. They constitute a group that requires special attention in relation to prevention, diagnosis and management [8]. Social distance program which are taken in the community as a result of efforts to control the disease, as well as the fear and panic of pregnant women from getting sick, lead to reduced referrals for prenatal care [1, 8]. Canceling hospital visits may reduce the probability of viral infection; the aftereffect could leave a bigger impact on pregnancy outcomes [13]. Pregnant women critically concern about the risk of infections, and high demand knowledge and measures on prevention and protection from COVID-19 [10]. A critical component in the management of any communicable disease threat is the care of vulnerable populations such as pregnant women [13]. So, Maternal care institutes should understand the demands and needs of pregnant women, optimize the means of prenatal care service, and provide tailored and accessible health education and service for the safety of them [8]. Pregnant women have the rights to participate in decisions involving their well-beings and what may or may not be done to their care. Understanding pregnant woman’s experiences and perspectives of prenatal care and factors affecting it’s during the outbreak of disease is particularly critical for enhancing the effectiveness of services delivery and addressing women’s needs and expectations. The mixed-method approach focuses on Epistemological Pluralism. As a result, it supports the combination of opinions, approaches, and different, even contradictory, methods if they are helpful for understanding concepts. The strategy proposed by this study may be helpful in weariness of health professionals and policy makers should be aware of factors influencing prenatal and self-care among pregnant women. So, it can improve prenatal care and is helpful in promoting health in pregnancy in critical duration of outbreak of disease. Therefore, it is hoped that the strategy proposed in the current study can lead to improve ability of accessible prenatal care.

## Data Availability

Not applicable.

## Abbreviations

COVID-19: Coronavirus disease 2019
FCV-19S: Fear of COVID-19 Scale
CDAS: Corona Disease Anxiety Scale
PSS: Perceived Stress Scale
